# Long and Accurate: How HiFi Sequencing is Transforming Genomics

**DOI:** 10.1093/gpbjnl/qzaf003

**Published:** 2025-02-07

**Authors:** Bo Wang, Peng Jia, Shenghan Gao, Huanhuan Zhao, Gaoyang Zheng, Linfeng Xu, Kai Ye

**Affiliations:** School of Automation Science and Engineering, Faculty of Electronic and Information Engineering, Xi’an Jiaotong University, Xi’an 710049, China; MOE Key Lab for Intelligent Networks & Networks Security, Faculty of Electronic and Information Engineering, Xi’an Jiaotong University, Xi’an 710049, China; Department of Gynecology and Obstetrics, Center for Mathematical Medical, The First Affiliated Hospital of Xi’an Jiaotong University, Xi’an 710061, China; School of Automation Science and Engineering, Faculty of Electronic and Information Engineering, Xi’an Jiaotong University, Xi’an 710049, China; MOE Key Lab for Intelligent Networks & Networks Security, Faculty of Electronic and Information Engineering, Xi’an Jiaotong University, Xi’an 710049, China; Department of Gynecology and Obstetrics, Center for Mathematical Medical, The First Affiliated Hospital of Xi’an Jiaotong University, Xi’an 710061, China; Department of Gynecology and Obstetrics, Center for Mathematical Medical, The First Affiliated Hospital of Xi’an Jiaotong University, Xi’an 710061, China; School of Automation Science and Engineering, Faculty of Electronic and Information Engineering, Xi’an Jiaotong University, Xi’an 710049, China; MOE Key Lab for Intelligent Networks & Networks Security, Faculty of Electronic and Information Engineering, Xi’an Jiaotong University, Xi’an 710049, China; School of Automation Science and Engineering, Faculty of Electronic and Information Engineering, Xi’an Jiaotong University, Xi’an 710049, China; MOE Key Lab for Intelligent Networks & Networks Security, Faculty of Electronic and Information Engineering, Xi’an Jiaotong University, Xi’an 710049, China; Department of Gynecology and Obstetrics, Center for Mathematical Medical, The First Affiliated Hospital of Xi’an Jiaotong University, Xi’an 710061, China; Faculty of Science, Leiden University, Leiden 2311 EZ, The Netherlands

**Keywords:** Long-read sequencing, Genome assembly, Complex genomic region, Variant detection, Centromere

## Abstract

Recent developments in PacBio high-fidelity (HiFi) sequencing technologies have transformed genomic research, with circular consensus sequencing now achieving 99.9% accuracy for long (up to 25 kb) single-molecule reads. This method circumvents biases intrinsic to amplification-based approaches, enabling thorough analysis of complex genomic regions [including tandem repeats, segmental duplications, ribosomal DNA (rDNA) arrays, and centromeres] as well as direct detection of base modifications, furnishing both sequence and epigenetic data concurrently. This has streamlined a number of tasks including genome assembly, variant detection, and full-length transcript analysis. This review provides a comprehensive overview of the applications and challenges of HiFi sequencing across various fields, including genomics, transcriptomics, and epigenetics. By delineating the evolving landscape of HiFi sequencing in multi-omics research, we highlight its potential to deepen our understanding of genetic mechanisms and to advance precision medicine.

## Graphical Abstract

**Figure:**
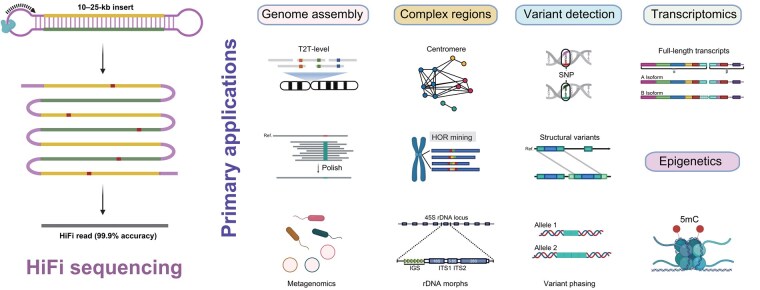

## Introduction

The complete genetic makeup of an organism, called its genome, holds crucial information for unraveling the origins and mechanisms of disease [1]. By enabling the identification of genomic regions associated with diseases, DNA sequencing technologies have provided invaluable insights into diagnosis, prognosis, and treatment strategies [2]. In this respect, next-generation sequencing (NGS) platforms are widely used because they produce short reads with relatively high accuracy, making them well-suited for detecting both single-nucleotide variants (SNVs) and small insertions and deletions (indels). However, NGS platforms struggle with tasks requiring longer stretches of DNA [3], such as *de novo* genome assembly, complex variant detection, and haplotype phasing. Long-read sequencing technologies, including PacBio continuous long read (CLR) sequencing [3] and Oxford Nanopore Technologies (ONT) sequencing [4], are better-suited for tackling these challenges as they routinely produce long reads exceeding 10 kb. These platforms work by directly sequencing single molecules, although compared to short-read NGS platforms they generally have reduced read accuracy (75% to 90%) [5]. Nevertheless, obtaining a completely accurate picture of the genome remains challenging, particularly for its most complex regions, such as centromeric sequences and tandem repeats [6,7].

**Figure 1:**
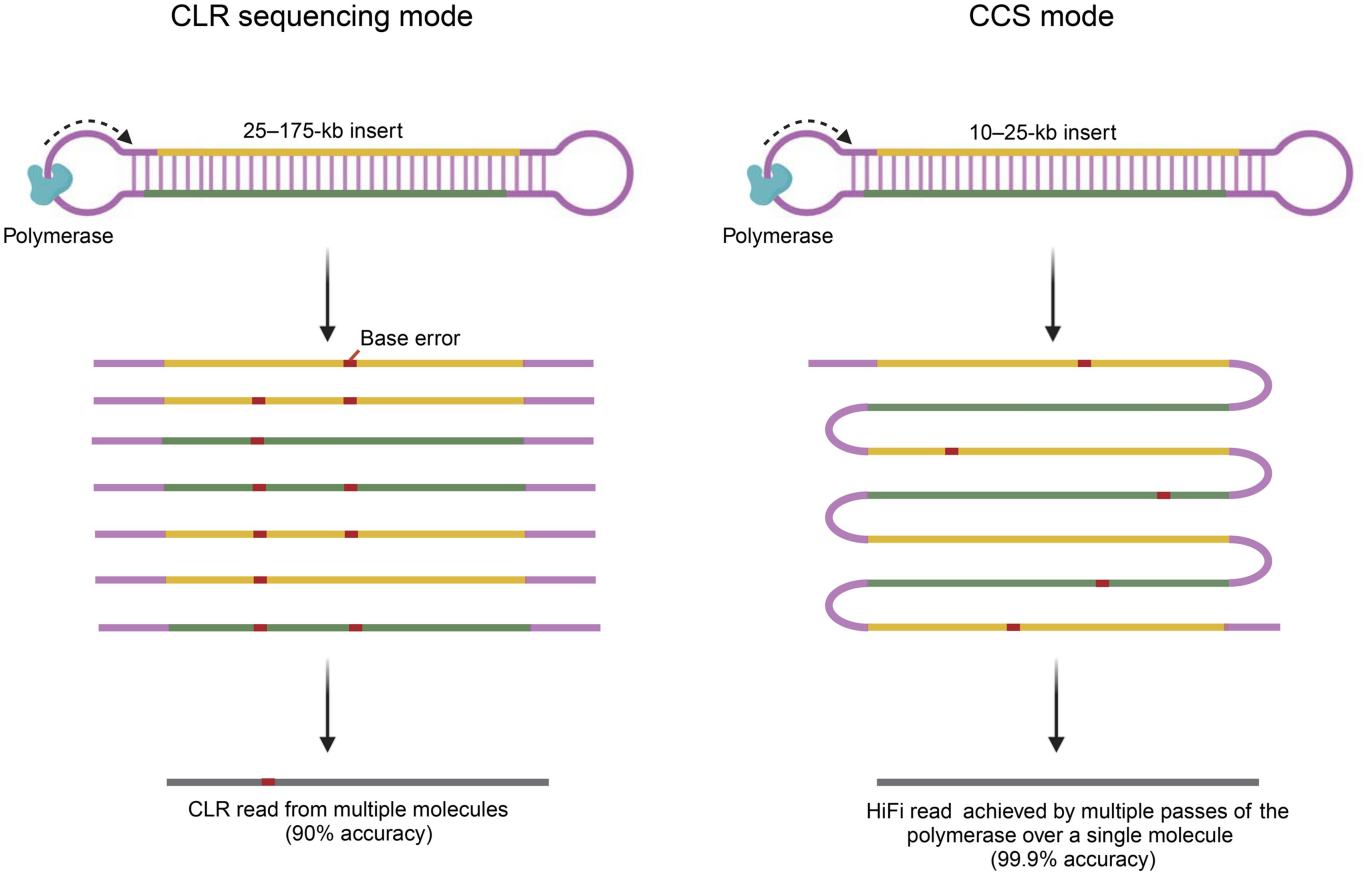
PacBio HiFi sequencing modes The PacBio platform produces long reads with two types of sequencing modes: CLR and CCS. CLRs, derived from 25–175-kb DNA fragment inserts, often exhibit an error rate of 8%–15% due to single-pass sequencing. By contrast, HiFi reads, generated from 10–25-kb inserts using CCS mode, have an error rate of ≤ 1% by leveraging multiple passes around the template. CLR, continuous long read; CCS, circular consensus sequencing; HiFi, high-fidelity.

In 2019, PacBio introduced high-fidelity (HiFi) sequencing technology, a significant advancement that addresses some of the limitations of previous technologies. HiFi implements circular consensus sequencing (CCS), which expands upon CLR sequencing by combining information from multiple readings of the same DNA molecule (discussed further below) [5]. CCS produces long, highly accurate reads from initially noisy individual subreads (**Figure 1**), and is currently implemented by the PacBio Sequel, Sequel II, and Sequel IIe systems. In 2023, PacBio released a new apparatus, Revio, which achieves a 15-fold increase in throughput which, coupled with a 4-fold decrease in cost, potentially enables the complete sequencing of approximately 1300 human genomes per year [8]. These advances have collectively been transformative for genomic research.

**Figure 2:**
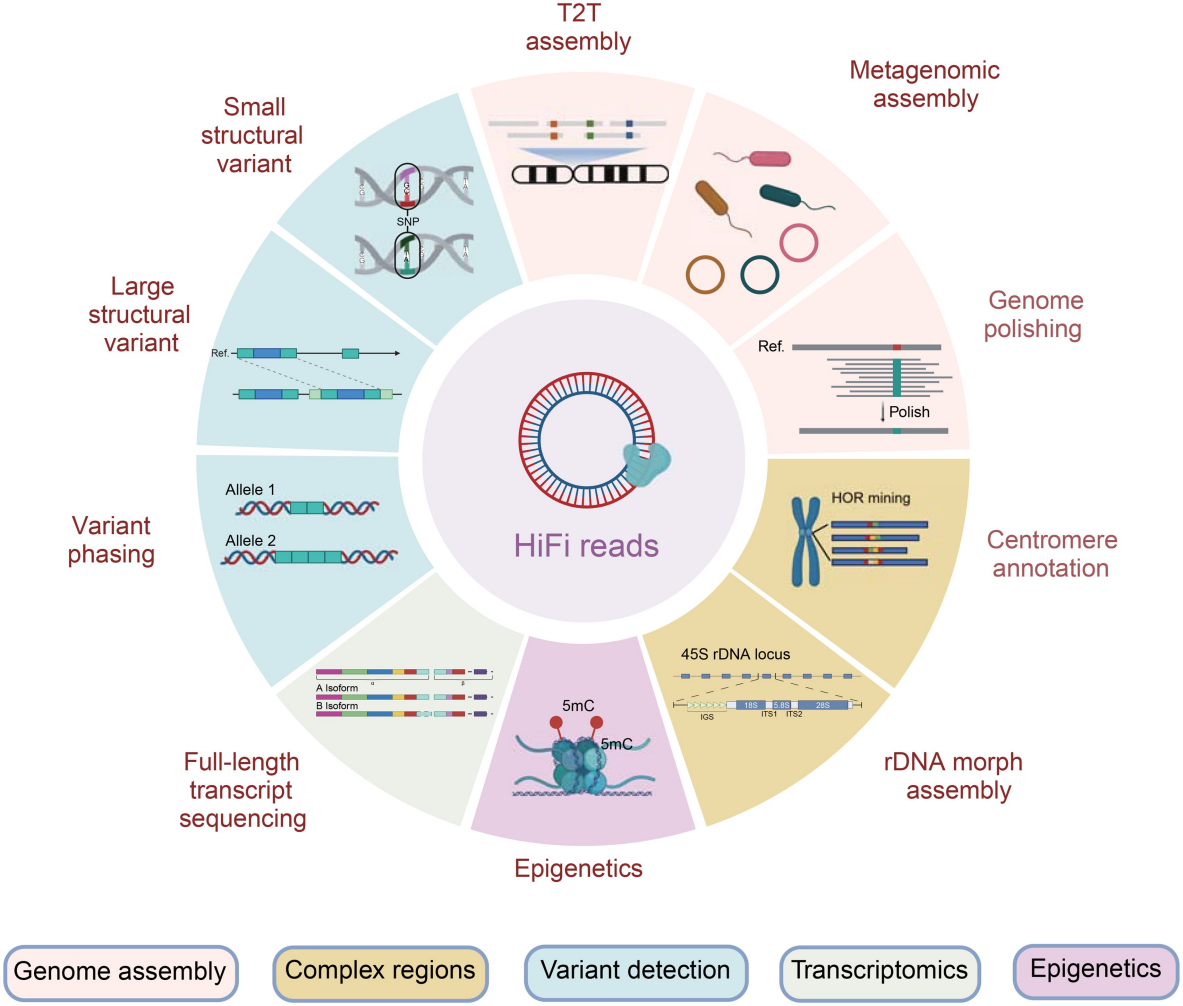
Primary applications of HiFi sequencing for genomic research The figure was created with BioRender.com. T2T, telomere-to-telomere; rDNA, ribosomal DNA; Ref., reference; SNP, single-nucleotide polymorphism; HOR, higher-order repeat; IGS, intergenic spacer; ITS, internal transcribed spacer.

In this review, we first provide an overview of the mechanisms of HiFi sequencing, and then discuss its applications across several different fields of research (**Figure 2**), the challenges faced, and the future prospects for this technology.

## HiFi sequencing mechanism and performance

The accuracy of HiFi sequencing lies in its iterative approach to crafting consensus reads, achieved through the repetitive sequencing of identical DNA fragments (Figure 1). This iterative cycle entails multiple traversals around the template, facilitated by enzymes exceeding the length of the DNA insert. Consequently, each DNA fragment undergoes multiple sequencing cycles, resulting in the accrual of data conducive to building a consensus sequence. Assembly algorithms ingest the data from each sequencing iteration, correct errors, and then construct a consensus mirroring the authentic DNA sequence [5].

A standout characteristic of HiFi sequencing is its capacity to produce elongated read lengths (typically 10 to 25 kb). It is worth noting that shorter DNA fragments could also generate CCS reads, but this would lead to reduced data production. Users should primarily focus on the quality control of read length. Furthermore, it achieves remarkable precision, with a median accuracy of 99.9% and accurate resolution of over 99.5% of homopolymers that are 5 or more bases in length [9].

In striking a balance between read length and accuracy, HiFi reads may effectively cover shorter repetitive sequences while simultaneously aiding in the identification of longer, more complex repeats (Figure 2). This capability is crucial for tasks like assembling complete genomes and detecting complex genetic variants associated with diseases (Figure 2).

## Genome assembly

### Single-species telomere-to-telomere assembly

HiFi sequencing’s ability to generate long, highly accurate reads has revolutionized the field of genome assembly. A major challenge in assembling complete genomes is resolving lengthy repetitive regions, such as centromeres. When combined with ONT ultra-long (> 100 kb) reads for hybrid assembly (discussed further below), HiFi can make a significant contribution toward producing completely gapless [telomere-to-telomere (T2T)] assemblies [10]. In 2020, HiFi sequencing played a key role in the first T2T assembly of a human X chromosome, primarily focusing on polishing, validating, and selecting unique anchors for the complex centromere region [11]. Two years later, the international T2T Consortium utilized both HiFi and ONT ultra-long reads to differentiate subtly diverged repeat copies, and ultimately assembled the 3.055 billion base pair sequence of a human genome [12], known as T2T-CHM13. This comprehensive sequence encompasses all centromeric satellite arrays, recent segmental duplications, and the short arms of all five acrocentric chromosomes. However, it is worth noting that CHM13 originates from a hydatidiform mole cell line with a nearly homozygous genome. This highlights the need for further advancements to assemble diploid genomes to the T2T level. We consider this imperative for clinically relevant samples and for enhancing our understanding of human genetic variation. To that end, Jarvis et al. evaluated methods for diploid human genome assembly and proposed an optimal combination of sequencing and assembly algorithms for accuracy and completeness of results with minimal manual curation [13]. They found that integrating both HiFi and ONT ultra-long data into a diploid assembly graph, along with long-range phasing information from trios, high-throughput chromosome conformation capture (Hi-C), or Strand-seq, could soon enable fully automated T2T diploid genome assemblies [14]. In 2023, two research teams from China accomplished two fully-phased T2T diploid genomes of a male Han Chinese, T2T-CN1 [15] and T2T-Yao [16], respectively.

HiFi sequencing has also been employed in the assembly of a number of both model and non-model T2T (or near T2T) plant genomes, including *Arabidopsis thaliana* [17–19], the moss *Physcomitrium patens* [20], the green alga *Chlamydomonas reinhardtii* [21], rice [22], maize [23], soybean [24], kiwifruit [25], Chinese cork oak [26], sorghum [27], four species of poppy (genus *Papaver*) [28], and two species of algae (genus *Chlorella*) [29]. Recently, in the animal kingdom, chickens [30], Chinese sea bass [31], geese [32], and finless porpoises [33] have also relied on HiFi sequencing to accomplish T2T-level genome assemblies.

To the best of our knowledge, there are at present 11 *de novo* assembly tools designed for HiFi reads, namely HiCanu [34], hifiasm [35], Flye [36], hifiasm-meta [37], metaFlye [38], PEREGRINE [39], Shasta with HiFi-mode [40], Verkko [14], MECAT2 [41], miniasm [42], and NextDenovo [43]. Yu et al. conducted an evaluation of the aforementioned 11 assemblers by using quality assessment tools and benchmarking universal single-copy orthologs, alongside several additional criteria [44]. The results indicate that hifiasm and hifiasm-meta emerge as the top choices for assembling both eukaryotic genomes and metagenomes with HiFi data. Verkko represents one of the initial attempts to automate T2T assembly utilizing HiFi data and ONT ultra-long reads (> 100 kb) [14]. However, Verkko demands significant computational resources and it lacks the capability to resolve haplotypes in polyploid samples. In contrast, Cheng et al. introduced hifiasm with ultra-long (UL) mode as an efficient solution for near T2T assembly of both diploid and polyploid samples [45]. hifiasm (UL) seamlessly integrates HiFi reads, ONT ultra-long reads, Hi-C reads, and trio data to generate high-quality assemblies in a single step. Notably, hifiasm (UL) combines two graphs — the first built from HiFi reads and the second from UL reads — resulting in a refined final assembly graph. Recently, Verkko v2.0 [14] or higher version was released, and it is significantly faster than the previous version. From the previous experience [15], for users with high-coverage HiFi reads, we suggest integrating Verkko with hifiasm for T2T assembly. This development marks a pivotal transition in T2T genomic research toward the era of pan-T2T genomics [45,46].

### Genome polishing

Although HiFi sequencing has facilitated T2T assembly, genome assemblies often still contain base-level errors, especially in homopolymer or low-complexity microsatellite regions, which are particularly susceptible to inaccuracies in HiFi reads [47]. Current genome polishing tools like Pilon [48], Racon [49], and NextPolish [50] often result in overcorrections and haplotype switch errors when rectifying errors in genomes assembled from HiFi reads [47]. To tackle this challenge, Hu et al. recently introduced NextPolish2 [47], a repeat-aware genome polishing tool proficient in correcting residual base errors in genomes assembled from HiFi reads. Compared to the modern polishing pipeline, Racon + Merfin [51], which was used to polish the CHM13 human T2T genome assembly, NextPolish2 minimizes excessive overcorrections, even in regions with highly repetitive elements [47]. The introduction of NextPolish2 holds significant promise for further enhancing the accuracy of T2T genomes.

### Metagenomic assembly

Metagenomic *de novo* assembly is a prevalent method for investigating microbial communities [52,53]. Prior to 2020, binning algorithms were frequently utilized to cluster short contigs for short-read metagenome assembly [54]. However, this method often led to highly fragmented and significant errors [55], thereby complicating or misguiding downstream analyses. The limitations of short-read metagenome-assembled genomes (MAGs) prompted the development of (among other algorithms) hifiasm-meta, harnessing the potential of HiFi reads for this task [37]. Through HiFi metagenomic assembly, they successfully obtained 102 complete MAGs from five human fecal samples, potentially representing complete genomes of human gut prokaryotic species. Furthermore, Zhang et al. enhanced microbial genomes and gene catalogs of the chicken gut through metagenomic sequencing of HiFi reads, recovering a substantial portion of novel genomes and genes previously overlooked in short-read-based metagenome studies [56]. Utilizing GTDB-Tk, all 337 species-level genomes were accurately classified at the order level; however, over half (*n* = 189) of these genomes failed to achieve species-level classification. In another study, Bickhart et al. reported lineage-resolved high-quality MAGs at the strain level in a complex metagenome [57]. By employing Hi-C binning and MagPhase phasing techniques, they were able to produce strain-level MAGs. These lineage-resolved complete MAGs represent a significant advancement toward achieving isolate-quality genome assemblies for complex microbial organisms. More recently, Benoit et al. have introduced a novel metagenomics assembler named metaMDBG designed specifically for HiFi reads [55]. The authors demonstrated that for intricate microbial communities, metaMDBG outperformed existing methods by yielding up to twice the number of high-quality circularized prokaryotic genomes. Additionally, metaMDBG exhibited superior capabilities in the retrieval of viruses and plasmids compared to other approaches [55].

## Resolving complex genomic regions

### Centromere

**Figure 3:**
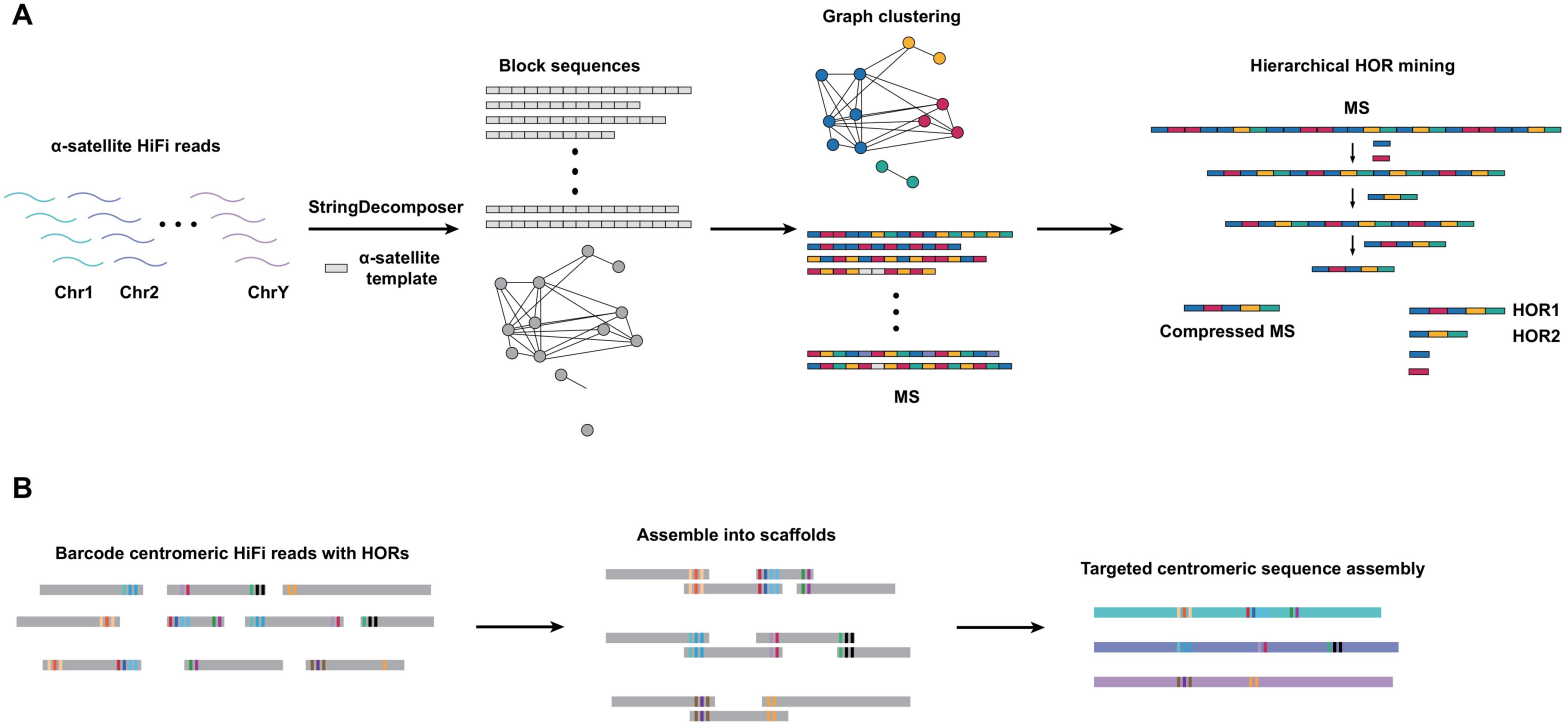
Applications of HiFi reads in centromere annotation and assembly **A**. Depicting a computational pipeline designed for annotating centromeric HOR patterns from HiFi reads. **B**. Barcoding the HiFi reads with centromeric HORs, followed by scaffolding and assembly of the centromeric sequences. MS, monomer sequence; Chr, chromosome.

Centromeres are crucial genomic regions and play a vital role in cell division. Understanding their structure is important for investigating how chromosomes are accurately copied and separated during cell division. Traditionally, analyzing centromeres has been challenging due to their repetitive DNA sequences, although HiFi reads have been instrumental in addressing the long-standing questions about their architecture and evolution [6]. Deep analysis of centromere architecture is critical for understanding genome stability, cell division, and disease development [58]. Centromere annotation refers to the process of partitioning centromeres into monomers and higher-order repeats (HORs). Annotation of HORs from HiFi reads is one direct way to obtain and validate centromere structures, for instance using Alpha-CENTAURI [59]. Due to the limitations of read-based annotation without genomic positional information for different centromeric HORs, researchers developed the first fully automated centromere annotation tool, HORmon, based on de Bruijn graphs and the T2T-CHM13 assembly [60]. In 2023, Gao et al. proposed a generalizable automatic centromere annotation tool named HiCAT [61], based on hierarchical tandem repeat mining. HiCAT employs a bottom-up iterative tandem repeat compression strategy to detect and represent locally-nested HORs. This approach significantly enhances annotation continuity and allows for the detection of fine structures and organization of HOR units, including length variations of HORs that were overlooked by HORmon. Gao et al. introduced an enhanced version of HiCAT, termed HiCAT-human [62], designed to automatically annotate centromere HOR patterns from HiFi reads (**Figure 3**A). HiCAT-human employs a combined approach integrating the “string decomposer”, “graph clustering”, and “hierarchical HOR mining” computational methods to annotate centromere sequences (Figure 3A). This analysis of HOR diversity can be utilized for tagging HiFi reads, aiding in further centromere targeted sequencing assembly (Figure 3B). Both HORmon and HiCAT series annotate centromeres based on known monomer sequences. In contrast, Wlodzimierz et al. introduced tandem repeat annotation and structural hierarchy (TRASH) [63], a tool capable of identifying and mapping tandem repeats in genome sequences without prior knowledge of repeat composition. TRASH proves particularly valuable for annotating putative centromeres in non-model species, where centromeres are comprised of unknown tandem repeat monomers.

### Ribosomal DNA morph assembly

Ribosomal RNA (rRNA) is a crucial component of ribosomes, with the genes encoding rRNA organized in repetitive clusters called ribosomal DNA (rDNA) arrays. For example, in humans, these arrays are located on chromosomes 13, 14, 15, 21, and 22, comprising a few hundred copies of an approximately 45-kb repeat unit arranged in tandem repeats [12]. A morph represents the sequence of one complete repeat unit appearing once or more times within the rDNA arrays. Detailed analysis of morph types, including chromosome-specific morphs, aids in assembling rDNA across different chromosomes. Rautiainen et al. developed a tool called Ribotin [64], which utilizes a combination of HiFi reads and ONT ultra-long reads to resolve variations among rDNA morphs. Ribotin effectively identifies the most abundant morphs in both human and nonhuman genomes. Importantly, for species with short rDNA morph sizes (up to 10 kb per morph), accurate morphs can be obtained using only HiFi reads. When integrated with the assembly tool Verkko, Ribotin enables researchers to accurately assemble rDNA morphs for each chromosome, providing a more complete picture of these important genes [64].

## Variant detection

HiFi sequencing technology has been widely applied to both healthy and diseased human samples, catalyzing the development of variant detection methods (**Table 1**). HiFi reads not only demonstrate high proficiency in detecting small variants but also promise performance in identifying larger structural variants (SVs) and tandem repeats, which are challenging for short reads and noisier long reads, respectively. Furthermore, using HiFi sequencing technology, heterozygous variants in diploid samples can now routinely be phased.

**Table 1:** List of variant detection methods for HiFi reads

Category	Method	Year	Link	Ref.
Small variant detection	DeepVariant	2018	https://github.com/google/deepvariant	[65]
	PEPPER	2021	https://github.com/kishwarshafin/pepper	[40]
	Clair3	2022	https://github.com/HKU-BAL/Clair3	[66]
SV detection	pbsv	2018	https://github.com/PacificBiosciences/pbsv	Not available
	Sniffles	2018	https://github.com/fritzsedlazeck/Sniffles	[67]
	Sniffles2	2024	https://github.com/fritzsedlazeck/Sniffles	[68]
	SVIM	2019	https://github.com/eldariont/svim	[69]
	cuteSV	2020	https://github.com/tjiangHIT/cuteSV	[70]
	SVision	2022	https://github.com/xjtu-omics/SVision	[71]
	SVision-pro	2024	https://github.com/songbowang125/SVision-pro	[72]
	DeBreak	2023	https://github.com/Maggi-Chen/DeBreak	[73]
	nanomonsv	2023	https://github.com/friend1ws/nanomonsv	[74]
Tandem repeat genotyping	adVNTR	2018	https://github.com/mehrdadbakhtiari/adVNTR	[75]
	tandem-genotypes	2019	https://github.com/mcfrith/tandem-genotypes	[76]
	Straglr	2021	https://github.com/bcgsc/straglr	[77]
	cTR	2023	https://github.com/morisUtokyo/cTR	[78]
	TRGT	2024	https://github.com/PacificBiosciences/trgt	[79]
	LongTR	2024	https://github.com/gymrek-lab/LongTR/	[80]
Assembly calling	SVIM-asm	2020	https://github.com/eldariont/svim-asm	[81]
	PAV	2021	https://github.com/EichlerLab/pav	[82]

*Note*: HiFi, high-fidelity; SV, structural variant.

### SNVs and indels

**Figure 4:**
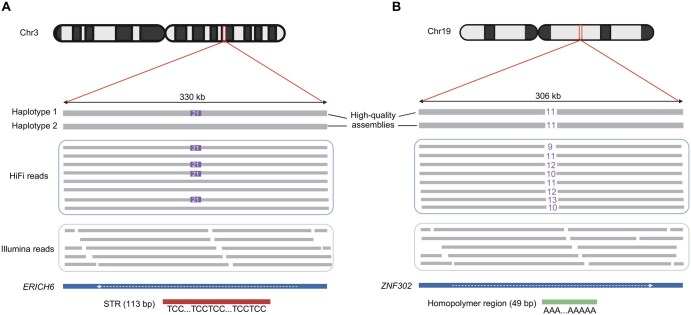
Variant detection of HiFi reads, short reads, and high-quality assembly in repeat regions **A**. Heterozygous insertion at a STR region with a TCC repeat. **B**. Homozygous deletion at a homopolymer region. STR, short tandem repeat.

SNVs and indels are the most abundant genetic variants in humans. In the last two decades, many small variants have been detected using short-read sequencing technologies, and genotypes associated with both pathogenic and other phenotypes have been identified through the efforts of many international consortia, including the 1000 Genomes Project [83], The Genome Aggregation Database (GnomAD) [84], and the Cancer Genome Atlas (TCGA) program [85]. However, these variants are mainly located at simple non-repetitive regions of the human genome, which account for approximately 85%–90% of the total sequence [83]. Prior to the release of T2T genomes, many SNVs and indels in complex regions had not been accurately resolved due to the inadequate length of sequencing reads [12]. Recently, HiFi reads achieved an F1 score of 0.998 for variant calling across all benchmark regions in the PrecisionFDA Truth Challenge, showing performance equivalent to short reads, which had an F1 score of 0.997 [86]. However, the longer length of HiFi reads enables more accurate alignments in genomic repetitive regions, where short reads may produce multiple aligned results [87]. For example, Jia et al. detected a 21-bp heterozygous insertion of a short tandem repeat (STR) region in *ERICH6* which was accurately identified by both haplotype-resolved assemblies and HiFi reads, but missed by Illumina short-read data [88] (**Figure 4**A). The authors highlighted an additional example involving a homozygous deletion within a homologous region (a 49-bp repeat of adenine) in *ZNF302*. This deletion was also overlooked by Illumina sequencing but identified as a homozygous deletion by HiFi sequencing and high-quality assemblies [88] (Figure 4B). Based on these results, haplotype-resolved assemblies identified the deletion as 11 bp in both haplotypes, while the deletion lengths detected by HiFi reads ranged from 9 to 13 bp. This suggests that haplotype-resolved assemblies might be more suitable for detecting small variants in homopolymer regions (Figure 4B). To that end, Vollger et al. systematically analyzed the small variants in segmental duplications (SDs) with HiFi assemblies, identifying a large number of SNVs in SDs that were previously considered largely inaccessible [89]. Furthermore, Chen et al. developed Paraphase to accurately identify variants of 160 long SDs in medically relevant genes which, also, were previously inaccessible [90].

Compared to germline variant calling, however, the detection of somatic variants in cancer samples requires much higher sequencing accuracy and depth due to their characteristically lower mutation frequency; this represents an ongoing challenge for HiFi sequencing.

### Large structural variants

SVs are typically described as gains, losses, and/or duplications of larger (≥ 50 bp) stretches of genome sequence. Although there are fewer SVs in a healthy human sample compared to small variants, the total length of the genome containing SVs is typically far greater [82]. Despite their large impact, SVs have been relatively understudied due to their complicated patterns compared to SNVs and indels. Nevertheless, long-read sequencing technology and algorithmic developments (Table 1) have facilitated the increased resolution of SVs in various samples. HiFi reads are able to cover most SVs and their high sequencing accuracy enables high-resolution identification of variant breakpoints, a capability that short reads and noisier long reads cannot achieve. Recent studies have demonstrated that HiFi reads can be used with deep learning models to analyze complex SVs involving multiple events [67,71]. Additionally, HiFi reads can detect mosaic and somatic variants in both mixed samples and cancer [68,72].

### Tandem repeat detection

Tandem repeats, including STRs and variable number tandem repeats (VNTRs), contribute a substantial number of genetic variants in the human genome [91,92]. Expansions and contractions of these repeats are associated with a number of diseases including Huntington’s disease and Fragile X syndrome [93]. Compared to short reads, HiFi reads can accurately resolve tandem repeat genotypes across the whole genome, particularly for VNTRs. Recently, advanced methods (Table 1), such as cTR [78], TRGT [79], and LongTR [80], have been developed for tandem repeat genotyping and visualization with HiFi sequencing reads, facilitating the application of this technology in tandem repeat-associated diseases [94,95]. Furthermore, HiFi reads are able to resolve some complex tandem repeats containing a mixture of multiple motifs, enabling deeper understanding of the relationship between the motif composition and phenotype [96,97].

### Variant phasing

**Figure 5:**
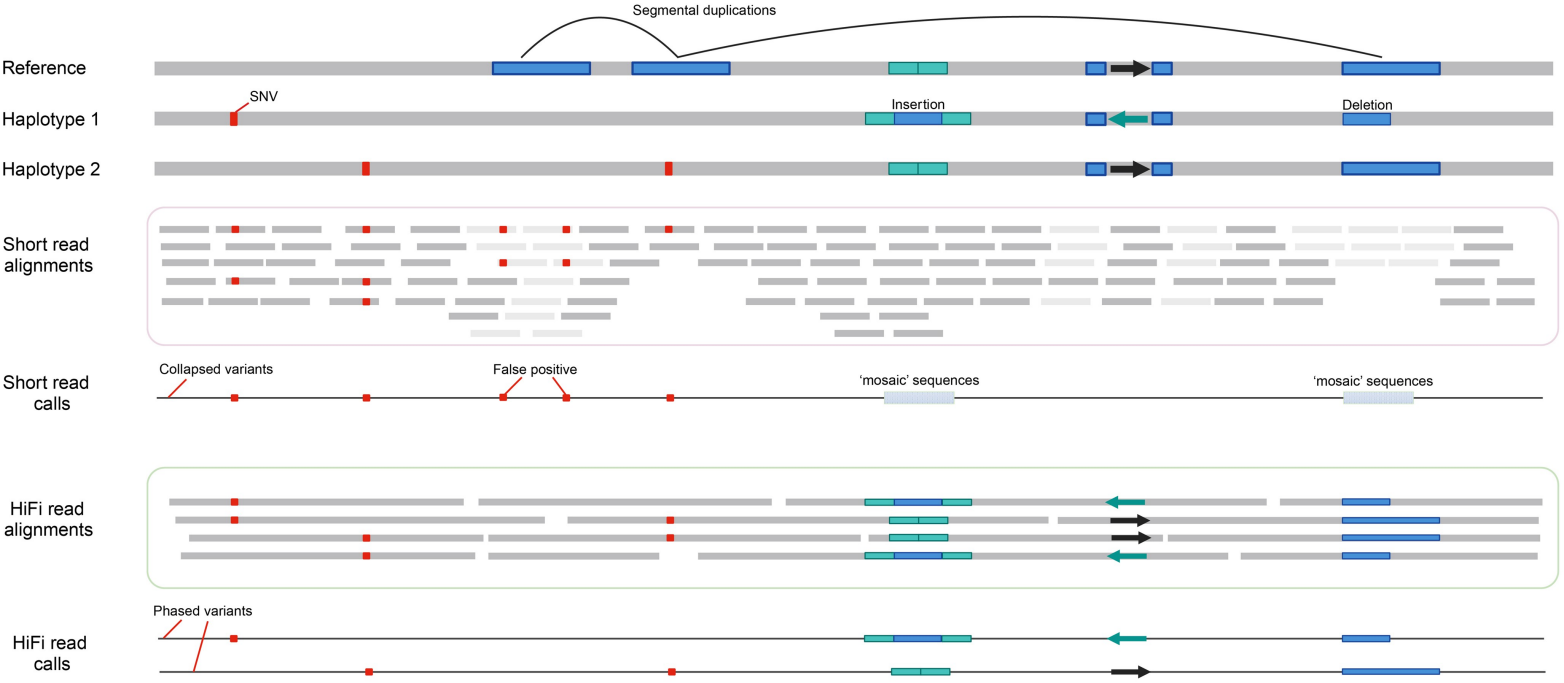
Advantages of HiFi reads in variant phasing In comparison to short reads, HiFi reads excel in the read-backed variant phasing category. Haplotype1 and haplotype2 are two haplotypes. SNV, single-nucleotide variant.

Variant calling using HiFi sequencing technology achieves the highest precision and recall across all variant categories, according to the PrecisionFDA Truth Challenge V2 [86]. By analyzing HiFi reads that span heterozygous variants (**Figure 5**), these variants can be phased with haplotype-resolved resolution [98–101]. HiFi sequencing captures a large number of informative bases overlapping with heterozygous variants, making it highly effective for identifying both small variants and SVs. This is particularly true in SD regions, where HiFi reads offer substantial advantages over short reads (Figure 5). Moreover, HiFi reads could be used to generate high-quality haplotype-resolved assemblies for diploid samples by advanced assemblies, such as hifiasm [35,45] and Verkko [14]. Based on HiFi assemblies, all scales of genomic variant test samples can be systematically categorized including variants larger than HiFi read lengths [82].

## Epigenetics

HiFi sequencing enables simultaneous calling of the four DNA bases and 5-methylcytosine (5mC) from untreated genomic DNA, allowing for genome-wide detection and phasing of genetic and epigenetic variants using a single, standard HiFi library preparation [102]. With the capability of long-read sequencing, one can achieve epigenetic analysis without the need for bisulfite treatment [103]. Unlike methods requiring chemical conversion of DNA, HiFi sequencing detects modifications in native DNA through impacts on base incorporation kinetics, ensuring high accuracy of sequence and methylation. Methylation detection with HiFi sequencing is highly consistent with bisulfite sequencing (average Pearson correlation = 0.97; mean absolute difference = 0.06) [104]. Additionally, HiFi sequencing provides access to the entire genome, including challenging regions like repeats and centromeres beyond the reach of short-read sequencing [105]. Moreover, HiFi sequencing facilitates phasing, allowing identification of allele-specific methylation arising from parental imprinting, genetic variation, or repeat expansions.

Recently, ccsmeth, a deep learning tool, was developed to detect 5mC methylation from HiFi sequencing by utilizing kinetic features [inter-pulse durations (IPDs) and pulse widths (PWs)] of HiFi reads at both the read and genome-wide site levels [105]. Recent studies have applied HiFi sequencing to call 5mC methylation specifically in the centromeric regions of genomes. For instance, in 2024, Wang et al. utilized ccsmeth to analyze methylation in the centromeric regions of the green algae genome and found that the centromeric CENH3 signals of green algae display a pattern of hypomethylation [29], similar to humans and higher plants. The pocket of hypomethylated CpG DNA was called the centromere dip region (CDR). Mastrorosa et al. employed HiFi and ONT ultra-long sequencing to fully assemble and extract methylation tags of the centromeres from a parent–child trio where the child presented with trisomy 21 [106]. The affected individual carries three distinct chromosome 21 centromere haplotypes, differing in length by 11-fold, with both the largest (H1) and smallest (H2) originating from the mother. The longest H1 allele exhibits a less clearly defined CDR based on CpG methylation and shows a significantly reduced signal in CENP-A chromatin immunoprecipitation sequencing (ChIP-seq) compared to H2 or paternal H3 centromeres. These epigenetic patterns suggest weaker kinetochore attachment for the maternally transmitted H1. Analysis of H1 in the mother indicates that the reduced CENP-A ChIP-seq signal, though not the CDR profile, existed prior to the meiotic nondisjunction event. These findings imply that recent differences in size and epigenetic characteristics of chromosome 21 centromeres may contribute to the risk of nondisjunction. HiFi sequencing has now become a comprehensive and accurate technology for 5mC detection and methylation phasing, particularly in repetitive genomic regions [104,105,107]. Its usage is expected to become even more widespread in epigenetics.

## Full-length transcript sequencing

HiFi sequencing holds the promise of significantly enhancing full-length transcript assembly. By generating long, accurate reads, it enables a more precise reconstruction of complex transcripts, thereby improving the identification of fusion genes and uncovering previously undetected isoforms with unprecedented accuracy [108,109]. This advancement not only aids in unraveling the intricate landscape of gene expression but also lays the groundwork for deeper insights into cellular functions and disease mechanisms.

Fusion genes, resulting from the fusion of two distinct pre-RNAs via *trans*-splicing, play a pivotal role in cancer development and progression. Detecting fusion transcripts accurately is crucial for cancer diagnosis, prognosis, and the development of targeted therapies. Long-read transcriptome sequencing has emerged as a powerful tool for identifying fusion genes, offering the ability to capture full-length transcripts and detect complex rearrangements with high sensitivity and resolution. Among the various tools available for detecting fusion genes in long-read transcriptome sequencing data, several stand out for their effectiveness and applicability across different cancer types. LongGF [110], JAFFAL [111], FusionSeeker [112], pbfusion [113], and CTAT-LR-fusion [114] are examples of such tools, each offering unique features and capabilities. Specifically, pbfusion, designed for isoform sequencing (Iso-Seq) HiFi data, has demonstrated its utility in identifying both known and novel fusion events in sarcomas. Its ability to accurately detect driver events highlights the reliability of Iso-Seq HiFi sequencing in uncovering clinically relevant fusion transcripts. By leveraging Iso-Seq HiFi data, pbfusion enables comprehensive characterization of fusion events, providing valuable insights into the molecular mechanisms driving cancer progression. Similarly, CTAT-LR-fusion offers a versatile solution for detecting fusion transcripts from long-read transcriptomic data, applicable at both bulk and single-cell levels. Its flexibility and scalability make it a valuable tool for studying fusion events across different cancer types and experimental settings. By integrating advanced algorithms and HiFi long-reads, CTAT-LR-fusion enables precise identification and characterization of fusion transcripts, facilitating the discovery of novel biomarkers and therapeutic targets. Compared to Nanopore direct RNA sequencing (DRS), Iso-Seq HiFi demonstrates higher precision in both exonic and intronic regions [112]. One drawback of HiFi sequencing, however, is that it typically produces fewer reads per run than ONT sequencing. Due to the limited throughput, multiplexed arrays isoform sequencing (MAS-Iso-Seq), a concatenation method designed to increase throughput by joining complementary DNA (cDNA) molecules into longer concatenated fragments, was introduced, increasing the throughput to nearly 40 million cDNA reads per run on the Sequel IIe sequencer [115]. Recently, PacBio has made significant advancements in yield, with the Revio system achieving approximately a 15-fold increase in throughput compared to the earlier Sequel IIe system. Additionally, tools like JAFFAL are proving valuable for enhancing full-length transcriptomic research [111]. Another limitation of HiFi sequencing is its primary application in detecting DNA modifications, whereas Nanopore DRS offers the advantage of direct, real-time sequencing of both DNA and RNA modifications [116].

Overall, the identification of fusion genes and novel isoforms through HiFi transcriptome sequencing data not only marks a substantial leap forward in cancer research but also holds immense promise for enhancing clinical diagnostics in the field.

## Single-cell sequencing

Understanding the intricate landscape of cancer requires comprehensive genotype–phenotype data at both the single-cell DNA and RNA levels. Single-cell NGS technologies emerged about a decade ago and have been commonly used for detecting variations through short-read sequencing protocols [117,118]. However, the limitations of short reads hinder their ability to capture the full range of genetic variation in individual cells.

To address this, Fan et al. developed single-molecule real-time sequencing of long fragments amplified through transposon insertion (SMOOTH-seq), a single-cell genome sequencing method based on HiFi long-read sequencing [119]. SMOOTH-seq reliably and effectively detects SVs and holds promise for *de novo* assembly of genomic DNA from individual cells [120]. However, SMOOTH-seq has limitations in detecting other types of genetic variations. To improve upon this, Hård et al. introduced a new method that uses an automated droplet-based multiple displacement amplification (dMDA) technique for single-cell genome assembly, combined with HiFi sequencing [121]. The inclusion of HiFi sequencing offers enhanced performance in detecting genetic variations in single cells, especially for haplotype phasing, complex SVs, and tandem repeats [121].

Single-cell transcriptome assembly may also benefit from full-length transcript sequencing. For example, HiFi long-read single-cell RNA sequencing (scRNA-seq) was conducted on clinical samples obtained from three ovarian cancer patients exhibiting omental metastasis [109]. Using HiFi sequencing depths of approximately 12,000 reads per cell, the authors captured a total of 152,000 isoforms, among which more than 30% were novel isoforms. In addition, cell type-specific isoforms and polyadenylation site utilization patterns could be identified in both tumor and mesothelial cells. Shi et al. developed HIT-scISOseq, a method designed to remove most artifactual cDNAs and concatenate multiple cDNAs for HiFi platforms, enabling high-throughput and high-accuracy scRNA-seq [122]. Building on this approach, Wang et al. adapted HIT-scISOseq for low-throughput cell analysis and applied it to sequence isoforms in single blastomeres of mouse preimplantation embryos [123]. Notably, the authors discovered 3894 transposable element loci that displayed dynamic changes during preimplantation development, which are likely involved in regulating the expression of neighboring genes.

Both ONT and PacBio technologies have been utilized in combination with scRNA-seq. Deng et al. conducted a systematic evaluation of scRNA-seq analysis performance using these two widely adopted long-read sequencing platforms [124]. In addition to enabling gene expression analysis, which is the primary application of NGS-based scRNA-seq, long-read scRNA-seq offers advanced capabilities such as gene splicing analysis and the identification of novel isoforms. Among long-read technologies, PacBio HiFi outperforms ONT in terms of sequencing quality, making it more effective for accurately identifying novel transcripts and detecting allele-specific gene and isoform expression [124].

## Comparative features and applications of HiFi and ONT sequencing

The comparative features and applications of HiFi sequencing and ONT sequencing highlight the strengths and trade-offs between these two state-of-the-art long-read sequencing platforms, such that users can make informed choices based on their individual needs (**Table 2**) [9,125]. HiFi sequencing utilizes CCS, achieving extremely high accuracy (Q33) through repeated reads of circular DNA templates. However, the accuracy of ONT (Q20) is lower, which may result in indels or other inaccuracies [126]. ONT Duplex sequencing reads have been reported to achieve an accuracy of up to Q30 [127,128]. However, it has not yet been commercialized, and we are looking forward to its development. HiFi offers moderate read lengths and excels in applications requiring high precision, such as initial assembly graph construction and phasing over heterozygous variants that are less than 10 kb apart [10]. In contrast, ONT sequencing is capable of generating ultra-long reads, often exceeding hundreds of thousands of bases or even a megabase, which helps compensate for the limitation in HiFi read length. ONT ultra-long reads are invaluable for resolving tangles and phasing through homozygous regions over 100 kb in length [10,45]. As such, HiFi and ONT ultra-long sequencing reads should undoubtedly complement each other in T2T genome assembly. Two widely used tools for co-assembling these data are Verkko [14] and hifiasm [45], as discussed above. HiFi sequencing is also typically used for detecting DNA 5mC modifications, whereas ONT provides direct, real-time sequencing of both DNA and RNA, and can detect a broader range of DNA modifications including 5mC, 5-hydroxymethylcytosine (5hmC), and *N*^6^-methyldeoxyadenosine (6mA) (Table 2).

**Table 2:** Summary of properties and applications of HiFi and ONT sequencing

Feature	HiFi sequencing	ONT sequencing
Technology type	CCS	Nanopore sequencing
Read length	Up to 25 kb (typically)	Over hundreds of thousands of bases long or even exceeding a megabase
Accuracy	Q33	∼ Q20 (up to Q30 for Duplex)
Read structure	Circular DNA template allows for multiple reads, generating a HiFi consensus	Linear DNA template read directly, producing single reads
Input	DNA	DNA, RNA
Typical runtime	24 h	72 h
Typical output file size (type)	55 GB (BAM)	1300 GB (fast5/pod5)
Detectable DNA modification	5mC	5mC, 5hmC, and 6mA
Variant calling	More accurate	Less accurate, inaccurately called indels
Chromosome-level genome assembly	Initial contig assembly combined with long-range data	Initial contig assembly with long-range data; polishing needed with HiFi or short-read data
T2T genome assembly	Initial assembly graph construction; phasing over heterozygous variants; limited in read length, need ultra-long reads	Resolving tangles; phasing through homozygous regions; need initial HiFi assembly

*Note*: ONT, Oxford Nanopore Technologies; CCS, circular consensus sequencing; T2T, telomere-to-telomere; BAM, Binary Alignment/Map; 5mC, 5-methylcytosine; 5hmC, 5-hydroxymethylcytosine; 6mA, *N*^6^-methyldeoxyadenosine.

## Remaining challenges and future perspectives

### Read length of HiFi data

Long-read sequencing offers unique advantages compared to short-read sequencing, with increased read lengths greatly benefiting genome assembly and the sequencing of complex regions such as centromeres, telomeres, rDNA regions, or large structural rearrangements. Currently, the two leading platforms for generating long-read data are PacBio and ONT, each with its own strengths. In general, HiFi technology yields sequences of higher accuracy compared to ONT, although ONT stands out both for its ability to produce ultra-long reads (up to 4 Mb) [129] and its scalability, ranging from small portable sequencers to larger benchtop instruments. In the field of personalized cancer genomics, there is a pressing demand for a bioinformatics algorithm that seamlessly integrates data from both the ONT ultra-long and HiFi sequencing platforms to compensate for the relative shortcomings in HiFi read lengths.

### Relatively high cost of HiFi sequencing

HiFi sequencing (Revio) is approximately four times more expensive compared to short-read sequencing platform (Illumina NovaSeq 6000 S4) [8,130]. The challenge presented by the high cost of HiFi sequencing is multifaceted. First, the significant initial investment required for equipment, reagents, and personnel training can serve as a significant barrier to entry for numerous research institutions and organizations. Furthermore, ongoing expenses related to maintenance, sample preparation, and data analysis compound the financial burden. Consequently, the steep price of HiFi sequencing limits access to cutting-edge genomic technologies, thereby constraining both the breadth and pace of scientific discovery and medical progress. Addressing this challenge involves exploring cost-saving strategies and enhancing efficiency in sequencing workflows, bolstering support for genomic research initiatives. One such strategy is targeted HiFi sequencing, which enables users to focus their sequencing efforts on specific genomic regions of interest. These regions may include intricate areas such as polymorphic human leucocyte antigen genes, centromeres, and VNTRs across the human genome. Another strategy to reduce costs is to increase throughput. PacBio users can attempt this by staying up to date with the latest single-molecule real-time (SMRT) cell versions, which offer substantial improvements. For example, the PacBio Revio system uses advanced SMRT cells with 25 million zero-mode waveguides, providing approximately a 15-fold increase in throughput compared to the earlier Sequel IIe system.

### Biases in library preparation

The throughput of HiFi sequencing is greatly influenced by molecular damage during library preparation. Therefore, it is crucial to develop appropriate protocols for extracting high-molecular-weight (HMW) DNA and methods for size selection. Accurate size selection of HMW DNA is vital for library preparation because long-read sequencing technology may exhibit bias toward sequencing smaller, more rapidly diffusing molecules [131]. This can have significant implications for downstream analyses, such as genome assembly and variant detection, where a comprehensive and unbiased view of the genome is essential. The bias toward smaller molecules not only affects the quality of the sequencing data but also the efficiency of the library preparation process. The presence of a bias can lead to an overrepresentation of certain genomic regions at the expense of others, which complicates the interpretation of sequencing results and potentially obscures biologically relevant information.

**Figure 6:**
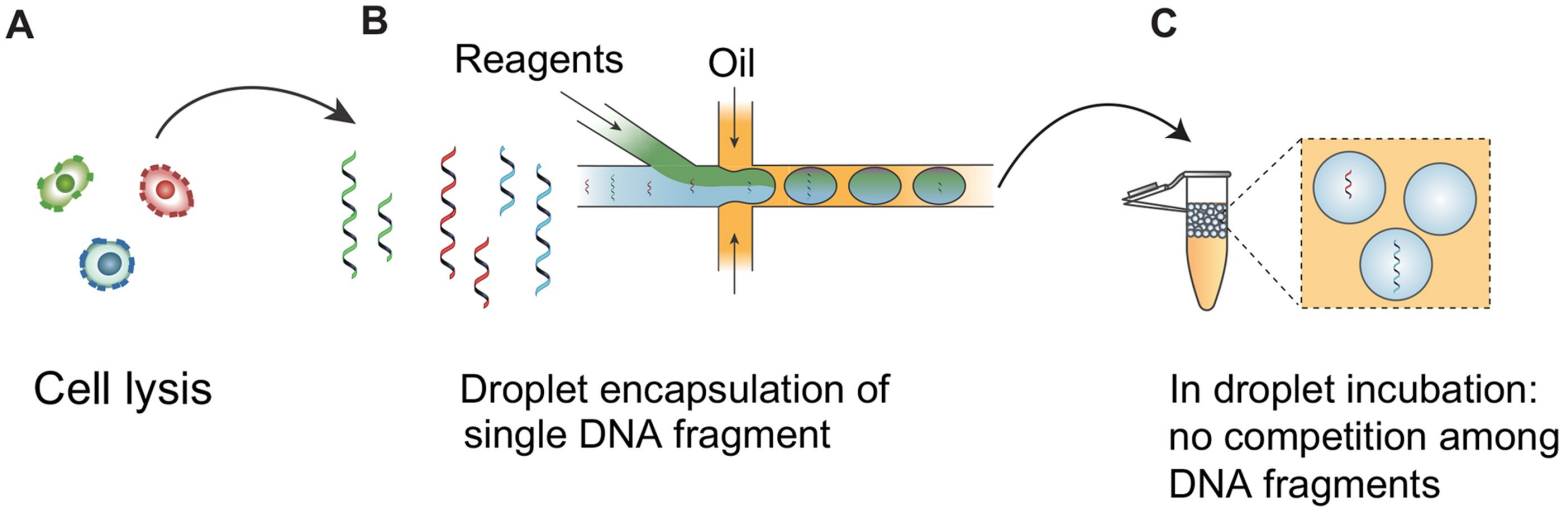
A typical workflow of droplet-based microfluidics for single DNA fragment experiment **A**. Cells are lysed and DNA fragments are collected. **B**. DNA fragments from cells and desired reagents are encapsulated into droplets with each droplet containing less than one DNA fragment. **C**. In droplet incubation to ensure there is no competition among DNA fragments.

Given these challenges, the development of innovative techniques, such as droplet microfluidics, is a significant step forward in addressing the issue of bias in library preparation for HiFi sequencing. Droplet microfluidics utilizes a large number of microfabricated droplets (tens of thousands to millions) with picoliter–nanoliter volumes [132]. As illustrated by **Figure 6**, each droplet encapsulates, on average, less than one DNA molecule, effectively eliminating competition between HMW and low-molecular-weight DNA fragments during critical library preparation steps such as ligation and polymerase chain reaction (PCR) [121,133]. Additionally, the increased DNA concentration within droplets compared to bulk solutions further enhances library preparation efficiency [132].

Droplet microfluidics further expands its utility in HiFi sequencing by enabling efficient targeted sequencing. Similar to digital droplet PCR (ddPCR) [134], each droplet can be compartmentalized with not only a single DNA molecule but also one or more fluorogenic probes specific to the target sequence. Following PCR amplification, droplets are analyzed for fluorescence, generating a binary positive or negative signal for target presence. Utilizing a fluorescent assisted droplet sorter (FADS) [135,136], positive droplets can be isolated with high throughput (> 90% accuracy) for downstream HiFi sequencing. Notably, to address potential sample limitations for HiFi sequencing, multiple displacement amplification (MDA) can be integrated within the droplet workflow to increase the total DNA content per droplet prior to sorting [121]. Furthermore, the utilization of clustered regularly interspaced short palindromic repeats/CRISPR-associated protein 9 (CRISPR/Cas9)-based [137] and hybridization-based enrichment strategies [138] represents progress toward enhanced and streamlined methodologies for targeted HiFi sequencing [8].

By overcoming these outstanding challenges, HiFi sequencing will gain even more value, providing the opportunity to delve deeper into genetic, epigenetic, and transcriptomic variations, along with their intricate connections to precision medicine and diagnosis.

### HiFi sequencing in precision medicine and diagnosis

HiFi long-read sequencing has emerged as a transformative technology in genomics due to its exceptional accuracy, long-read capabilities, and comprehensive coverage of complex regions in the genome. The fields of precision medicine and personalized diagnosis increasingly require highly accurate genomic data to identify mutations, SVs, and other genetic anomalies associated with an individual’s response to disease [139]. By broadening the spectrum of detectable variants to more complex genomic regions, HiFi sequencing can improve the accuracy of diagnosing genetic diseases, identifying disease subtypes, and tailoring personalized treatment regimens for conditions like cancer, rare genetic disorders, and cardiovascular diseases [109,140]. Despite the diverse applications of HiFi sequencing technologies, their routine use in clinical settings nevertheless faces unique challenges [139]. One of the key obstacles is assembling complete cancer genomes to fully capture and resolve complex genetic variations, such as SVs and large rearrangements [141–143]. Additionally, creating personalized pangenome references that incorporate functional information, including cellular epigenetics and transcriptomic changes, remains an important goal for more comprehensive analysis [144–146].

To overcome these challenges, it is essential to improve the stability and reliability of cancer sequencing technologies. Furthermore, advancements in bioinformatics algorithms are needed to better analyze and interpret the vast amounts of data generated. Successfully integrating these developments into precision medicine and diagnostic laboratories will be critical for realizing the full potential of HiFi sequencing in clinical practice.

## CRediT author statement

**Bo Wang:** Investigation, Writing – original draft, Visualization, Funding acquisition. **Peng Jia:** Investigation, Writing – original draft, Visualization. **Shenghan Gao:** Investigation, Visualization. **Huanhuan Zhao:** Investigation, Visualization. **Gaoyang Zheng:** Visualization. **Linfeng Xu:** Investigation, Writing – original draft, Visualization. **Kai Ye:** Conceptualization, Supervision, Writing – review & editing, Funding acquisition. All authors have read and approved the final manuscript.

## Competing interests

The authors have declared no competing interests.
